# Gender differences in presbyopia

**Published:** 2009-06

**Authors:** Ilesh Patel, Sheila West

**Affiliations:** Research Fellow, Dana Center for Preventive Ophthalmology, Wilmer Eye Institute, Johns Hopkins University, 600 N Wolfe St, Baltimore, MD, USA.; El-Maghraby Professor of Preventive Ophthalmology, Dana Center for Preventive Ophthalmology, Wilmer Eye Institute, Johns Hopkins University, 600 N Wolfe St, Baltimore, MD, USA.

**Figure:**
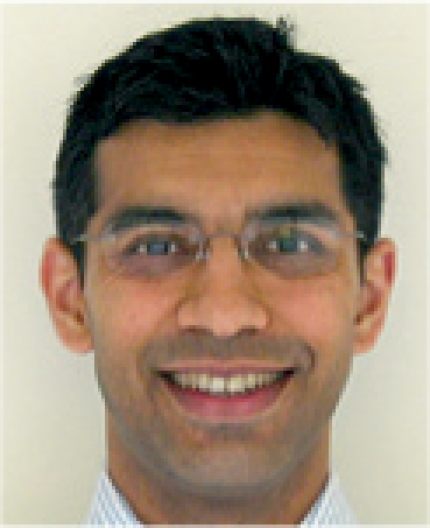


**Figure:**
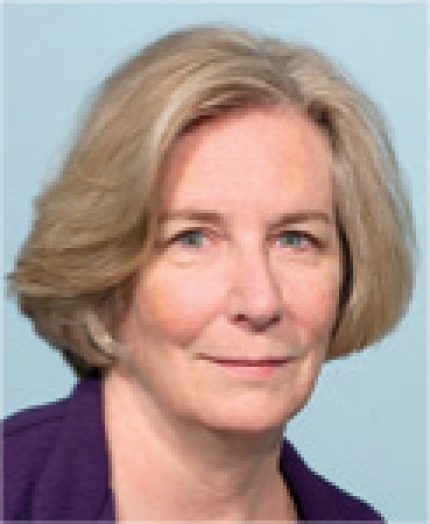


Presbyopia is the loss of lens accommodation with age that results in an inability to focus at near distances. It is receiving growing attention because of the recognition that good near vision is needed to accomplish a broad range of tasks, not only reading and writing.

Research shows that there are differences between men and women in the prevalence, age of onset, and severity of presbyopia,^1^ in the types of tasks for which men and women use near vision,^2^ and in the ability of men and women to afford spectacles for correction of presbyopia.^3^

## Prevalence, age of onset, and severity

The prevalence of presbyopia in low- and middle-income countries is not well known, but several studies have indicated that the prevalence is higher among women:

- Morny, using hospital chart reviews, found a prevalence of 65 per cent in Ghanaian women.^4^
- In southern India, Nirmalan et al. found a prevalence of 55 per cent in subjects aged 30 years and older.^5^ The prevalence of presbyopia increased with increasing age and women had 40 per cent higher odds of being presbyopic.
- Duarte et al. in Brazil estimated the prevalence of presbyopia in 3,000 adults 30 years and older at 55 per cent.^6^ Once again, age and female sex were associated with higher prevalence.
- Age-adjusted data collected during the authors' study of 1,709 people aged 40 and above in rural Tanzania showed higher prevalence among women than men (Figure 1). In multivariate analysis, women had 46 per cent higher odds of being presbyopic (defined as the ability to read N8 at 40 cm using a logMar E chart).^1^

**Figure:**
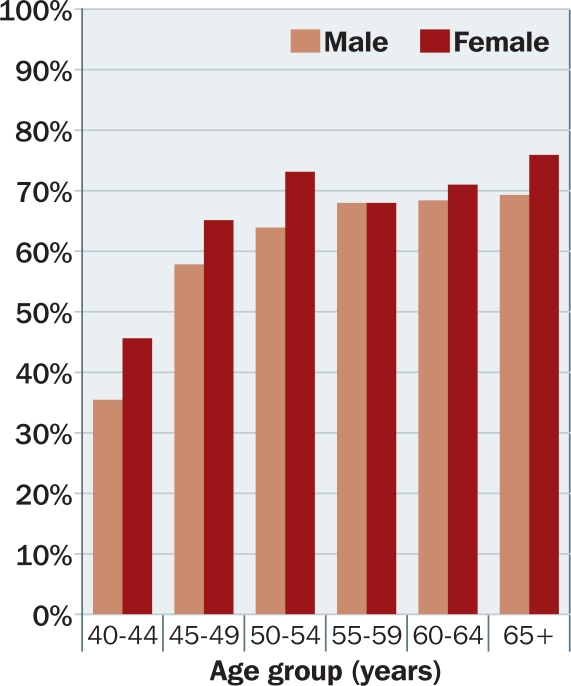
Figure 1. Prevalence of presbyopia by age and gender in a rural Tanzanian population^1^

**Figure:**
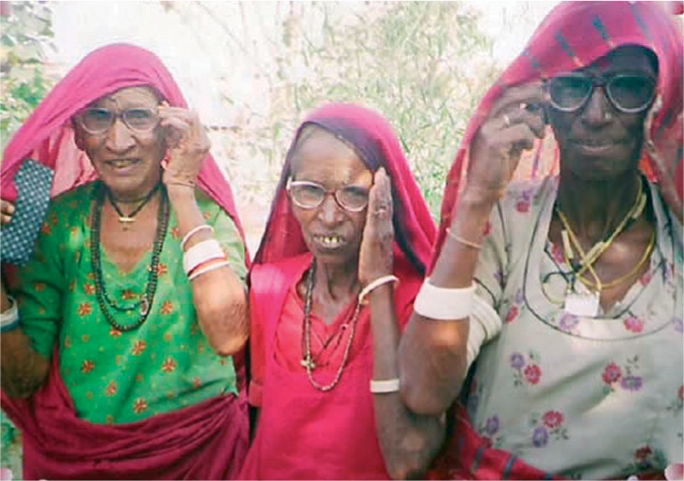
Outreach camps are one way to reach women who need near vision spectacles. INDIA

Prakash Tyagi, GRAVIS

Pointer, in his clinic-based study, observed that presbyopia affected women earlier than men.^7^ Our study showed that women also had more severe presbyopia than men across all age groups.^1^

## Impact on women

The authors' study in Tanzania showed that, in rural communities where near vision tasks other than reading and writing are predominant, uncorrected presbyopia had a substantial impact on quality of life.^2^

We found that women used near vision for lighting and adjusting lamps, winnowing grain, sorting rice, weeding, sewing, cooking food, and dressing children. Men reported using near vision for lighting and adjusting lamps, reading, writing, harvesting, and weeding. Nearly 80 per cent of people with presbyopia reported having problems with near vision and 71 per cent were dissatisfied with their ability to do near work. Women were just as likely as men to report problems. No other studies to date have examined the tasks people use near vision for in rural settings.

## Intervention

Spectacles offer a safe, effective, and economic option for the correction of presbyopia. However, there is little research on the determinants of, and barriers to, the use of near-vision spectacles.

Only six per cent of the participants with presbyopia in our study in Tanzania had corrective spectacles. Almost all these participants were men.

In Timor-Leste, among those who were presbyopic, 31 per cent of men and 21 per cent of women had spectacle correction.^8^

In our study in Tanzania, a high proportion of participants (69 per cent) were able to afford spectacles at a price that covered the cost and shipping of the spectacles. Men were more likely to be able to afford spectacles, whereas a higher proportion of women needed to rely on another person to help them afford spectacles.

In Timor-Leste, 25 per cent of men compared to 15 percent of women were willing to pay US $3 for spectacles (age-adjusted prevalence).^8^

The majority of participants in our study in Tanzania did not know where to get spectacles. Women were less likely to know than men. Among those (both men and women) who knew where to go, a third could not afford the means to travel to a location where spectacles could be obtained. Once again, women were less likely to be able to afford the travel.

## Conclusion

Women have a higher prevalence of, and more severe, presbyopia. Despite this, women in low- and middle-income countries are less likely to have spectacle correction. Men and women have different needs for near vision but are equally likely to report problems with daily activities due to near vision impairment. However, women are less likely to be able to afford correction and less likely to know where to get spectacles. These gender differences represent additional challenges for presbyopia correction programmes.
